# Neurosurgical content for mobile devices

**DOI:** 10.4103/2152-7806.68932

**Published:** 2010-09-01

**Authors:** Pieter L. Kubben

**Affiliations:** Department of Neurosurgery, Maastricht University Medical Center, Netherlands

Moore’s law is long-term trend in the history of computing hardware. It basically says that the number of transistors that can be placed inexpensively on an integrated circuit roughly doubles every 2 years.[2] Although the exact time frame varies in several documents, the description originates from 1965 and is still valid today. Modern computers have tremendous processing power, but at the same time they are a fraction of the size compared to their predecessors of 20-30 years ago. Modern computing hardware shows an increasing trend toward mobility, while offering high resolution color displays, multimedia capacities, and integrated network connectivity (phone, internet, GPS, Bluetooth). The latter is particularly important for developing countries, where cable networks are rare. Satellite-based wireless networks for phone and Internet are expected to become the primary choice for connectivity, and mobile devices can serve as a cost-effective hardware solution.

To have users benefit from all features on mobile devices, content needs to be optimized for smaller screens and limited keyboard input. This is also important for scientific and clinical applications. Mobile applications are used differently from desktop applications. They should allow users to perform some common tasks in a quick and efficient manner. Of course, the nature of these tasks depends on the nature of the application. For *Surgical Neurology International* (SNI), we wanted a web application that offers you quick access to the pillars of our website: the articles, the posts and the forum. It is also important that you are able to qucikly and easily access our multimedia and social network updates.

## SNI MOBILE

**Figure 1:**
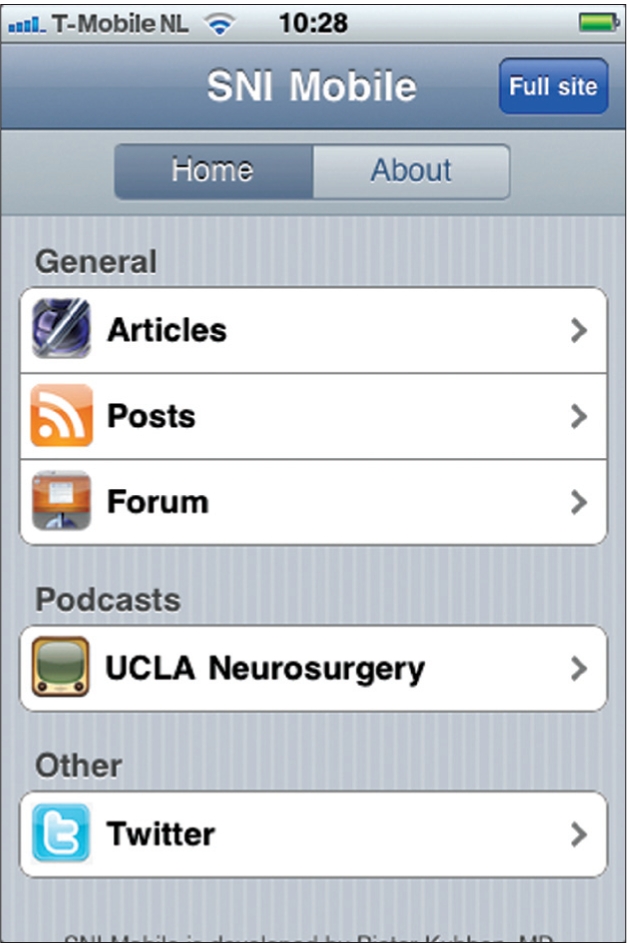
SNI mobile homepage

**Figure 2:**
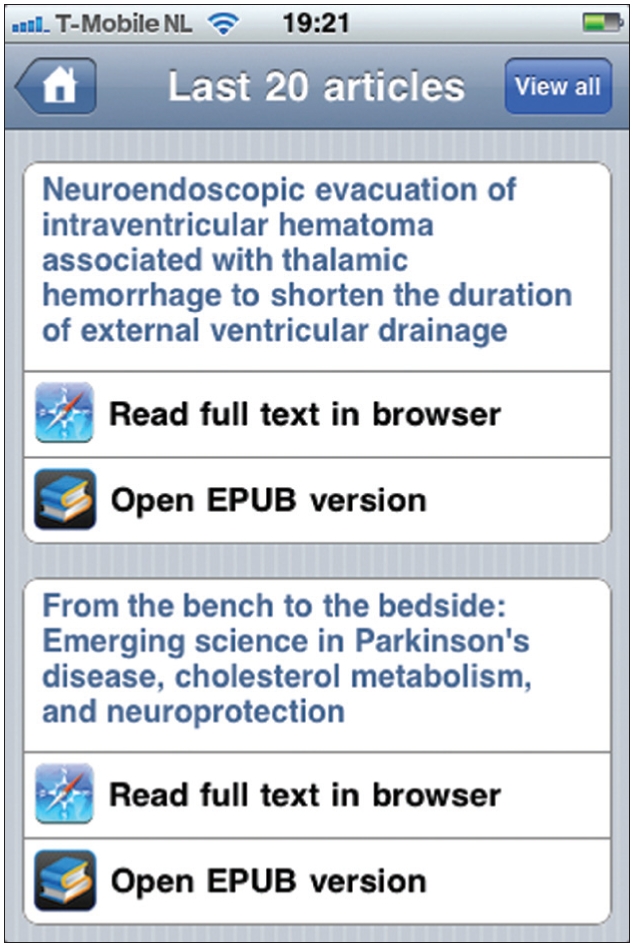
Recent articles with direct access to full text

Our web application, “SNI Mobile,” does just that: it offers you quick access to the latest articles, posts, forum items, Twitter updates and podcasts [Figure 1]. By selecting a category, you get the titles for the last 20 items of that category in reverse chronological order [Figure 2]. If you select an item from the podcasts category, the web application will automatically open iTunes if you access it from iPhone, iPod Touch or iPad. Although the layout of the web application has been optimized for these devices, it can be accessed from any device (like Blackberry, Android, Windows Mobile or Symbian-based). Add this link to your mobile browser’s favorites to access the web application quickly: www.surgicalneurologyinternational.com/mobile

Alternatively, go to our website and select “Neurosurgery 2.0” > “Mobile” in the menu for further instructions.

## ARTICLES IN EPUB FORMAT

**Figure 3:**
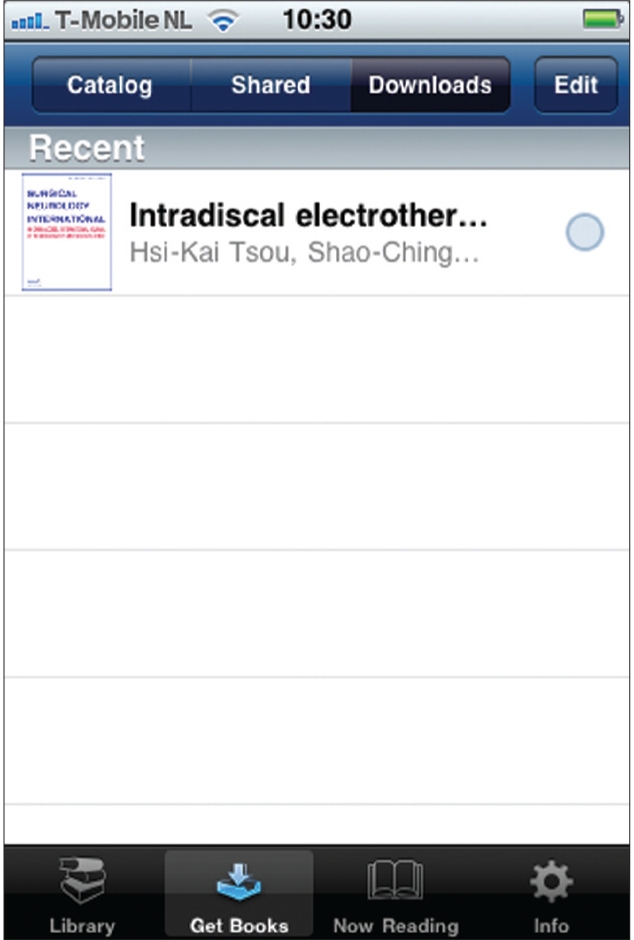
Screenshot from stanza on iPhone

**Figure 4:**
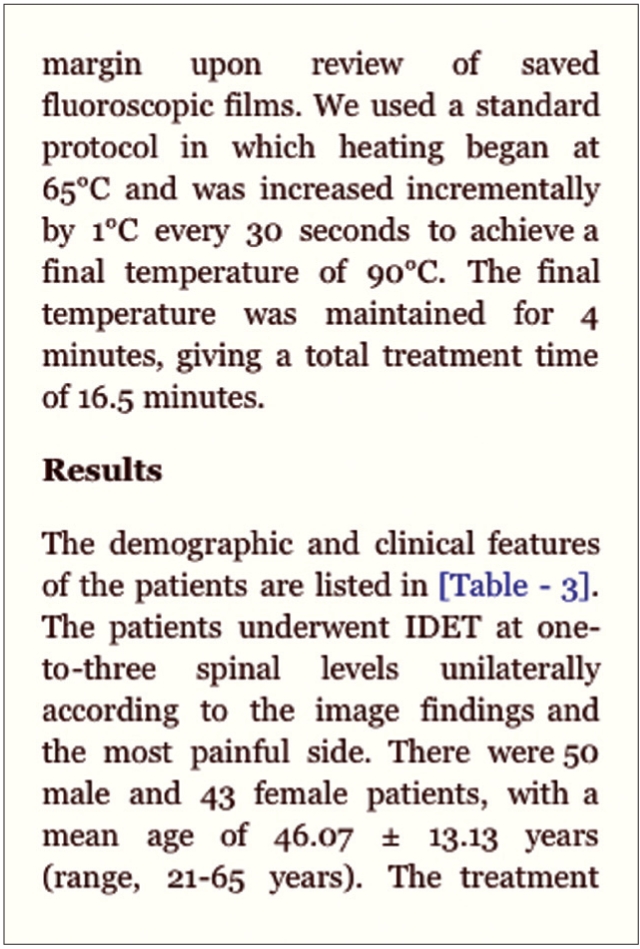
Reading an article in stanza on iPhone

You are probably familiar with downloading articles in Portable Document Format (PDF). The main advantage of PDF files is that they look the same on all computers, regardless of what text editor is used, which fonts are installed, or which printer settings are chosen. There are many good free PDF viewers available, and for desktop computers this format became the standard for downloading scientific articles. The main disadvantage for using them on mobile devices is that the original page size is maintained. Therefore, reading PDF files on a small screen can be a cumbersome task. This problem can be solved by using the EPUB format, which is a free and open e-book standard for electronic publication (hence the name).[1] There are EPUB readers for many different mobile devices, and they allow the user to increase or decrease the font size to optimize reading on the small screen. Stanza is a free EPUB reader for iPhone, iPod Touch and iPad that I can recommend [Figure 3]. Good suggestions for other devices are always welcome. Figure 4shows an example of how text may look on a small screen.

## EPUB AND OPEN ACCESS

As SNI is an open-access journal, all full-text articles can be downloaded for free, without any form of registration required. This is especially useful when it comes to downloading articles on mobile devices. There is no need to be connected to your hospital or university network; you can download EPUB versions from all our articles. This can be done from our website, or directly from the web application [Figure 2].

## NOW WHAT?

The foundation of every scientific journal is formed by (peer-reviewed) articles. This editorial explains how mobile devices can be used to access SNI’s content, and how the EPUB format facilitates reading articles on small screens. SNI Mobile is a web application that allows quick access to the journal’s main categories, with a layout that is optimized for iPhone and related devices. We intend to use this web application as a starting point for further mobile development. Based on our experience and your feedback we will continue working on separate applications for iPhone, iPad, Android, and maybe Blackberry in the future. We would like you to read not only our articles, but also our (blog) posts and forum items. Also, we would like to offer you our multimedia on mobile devices, starting with some podcasts on basic and clinical neurosurgery.

What sorts of applications and content would you be particularly interested in?
